# Second primary cancers in patients with a pharyngeal index tumour: a register-based cohort study

**DOI:** 10.1186/s12885-024-13103-x

**Published:** 2024-11-11

**Authors:** Rayan Nikkilä, Elli Hirvonen, Aaro Haapaniemi, Janne Pitkäniemi, Nea Malila, Antti Mäkitie

**Affiliations:** 1 Department of Otorhinolaryngology – Head and Neck Surgery, University of Helsinki, Helsinki University Hospital, P.O. Box 263, Helsinki, FI-00029 HUS Finland; 2 Research Program in Systems Oncology, Faculty of Medicine, University of Helsinki, Helsinki, Finland; 3 Finnish Cancer Registry, Institute for Statistical and Epidemiological Cancer Research, Helsinki, Finland; 4 Department of Oral and Maxillofacial Surgery, Päijät-Häme Joint Authority for Health and Wellbeing, Lahti Central Hospital, Lahti, Finland; 5 Unit of Health Sciences, Faculty of Social Sciences, Tampere University, Tampere, Finland; 6 Department of Public Health, University of Helsinki, Helsinki, Finland; 7 Division of Ear, Nose and Throat Diseases, Department of Clinical Sciences, Intervention and Technology, Karolinska Institutet and Karolinska Hospital, Stockholm, Sweden

**Keywords:** Head and neck cancer, Pharynx, Nasopharynx, Oropharynx, Hypopharynx, Second primary, Carcinoma

## Abstract

**Background:**

While prior research on the SPC (second primary cancer) risk among pharyngeal carcinoma (PC) patients has been conducted in other regions, the European perspective is underrepresented. Our register-based cohort study aims to assess the subsite-specific risk of SPC among individuals initially diagnosed with a pharyngeal index tumour.

**Methods:**

Standardized incidence ratios (SIR) of SPC were calculated relative to the general population for all patients diagnosed with a primary oropharyngeal, nasopharyngeal, and hypopharyngeal carcinoma (OPC, NPC, and HPC) in Finland during 1953–2021.

**Results:**

A total of 4701 PC patients – 3320 men (71%) and 1381 women (29%) – were identified. The average and median follow-up times were 5.7 and 2.8 years, respectively. A SPC was diagnosed in 561 patients (11.9%): in 12.3% of men (*n* = 410) and 10.9% of women (*n* = 151). For male PC patients, the overall SIR for an SPC at any primary site was 1.83 (95% CI: 1.65–2.01). For female patients, the corresponding SIR was 1.89 (95% CI: 1.60–2.22). OPC and HPC showed increased risks for SPCs of the mouth/pharynx (SIR 4.41 and 6.91, respectively) and respiratory organs (SIR 3.51 and 4.80). OPC patients also had an increased risk in digestive organs (SIR 1.83). Male NPC patients exhibited increased risks for oral/pharyngeal, brain, and haematolymphoid SPCs (SIRs 5.14, 6.60, and 3.05, respectively).

**Conclusion:**

PC patients face an 80% higher SPC risk, which persists decades after treatment. Healthcare professionals must be aware of this, providing counselling and encouraging a healthy lifestyle, including smoking cessation, while monitoring symptoms.

## Background

Pharyngeal carcinoma (PC) encompasses malignant tumours affecting the oropharynx, hypopharynx, and nasopharynx, with squamous cell carcinomas (SCCs) constituting over 90% of all cases [1]. According to the Global Cancer Observatory database (www.gco.iarc.fr), over 300 000 cases of PC were diagnosed globally in 2020 and the number of new cases is anticipated to increase by over 40% by year 2040. However, incidence rates vary markedly worldwide, especially for nasopharyngeal and oropharyngeal carcinomas (NPCs and OPCs, respectively), with the latter being more prevalent currently in high-income countries. Namely, while approximately 80% of new cases of NPC are recorded in Eastern and South-Eastern Asia, it is rare in Europe and Northern America where less than 6% of all new cases are diagnosed. In contrast, approximately 45% of OPC cases are nowadays diagnosed in Northern America and Europe.

Patients with head and neck cancer (HNC) have been proven to carry an increased risk of second primary cancers (SPCs). These cancers arise especially in the upper aerodigestive tract [2, 3], possibly due to the field cancerization effect induced by the chronic use of tobacco and alcohol [1, 4]. Still, though commonalities exist among HNCs, including the role of tobacco and alcohol in many cases, PCs stand apart due to their distinct risk profiles such as viral infections (Epstein-Barr and human papillomavirus [HPV]), dietary practices, genetic predisposition, specific environmental exposures. Indeed, while Epstein-Barr virus infection along with genetic predisposition, diets high in nitrosamine-rich salted fish and meats – common in Southeast Asia – and wood dust have been associated with NPC [5–8], the high incidence of OPC in Europe and Northern American has been attributed to a surge in the proportion of HPV-driven tumours [9].

While prior research on SPC risk among PC patients has been conducted in other regions, follow-up time remains frequently limited to less than 10 years and the European perspective has been notably underrepresented. Large patient cohorts have primarily been studied in the United States and Asia, and even on a global scale, there are limited studies that separately examine subsite-specific risk and trends over time [2, 3, 10]. Our register-based cohort study aims to fill this gap by assessing the subsite-specific risk of SPC among individuals initially diagnosed with a first primary PC in Finland between 1953 and 2021. To the best of our knowledge, our study, with 4700 PC patients, represents one of the largest cohorts of PC patients published in Europe with over 10 years of complete follow-up.

## Patients and methods

*Population.* Data on patients diagnosed with PC in Finland between 1953 and 2021 were obtained from the Finnish Cancer Registry (FCR). The FCR includes all new primary cancer cases diagnosed in Finland since 1953, providing comprehensive information on the primary site, histology, and complete follow-up until death or emigration. ICD-O-3 topographical and morphological codes (available at http://www.iacr.com.fr/index.php?Itemid=577) were employed to extract data of patients diagnosed with PC: neoplasms of any histology occurring in the oropharynx (C01.9, C02.4, C05.1, C05.2, C09.0, C09.1, C09.8, C09.9, C10.0, C10.2-C10.4, C10.8, C10.9), nasopharynx (C11.0-C11.3, C11.8, C11.9), hypopharynx (C12.9, C13.0-C13.2, C13.8, C13.9), and at an unspecified location in the pharynx (C14) were included. Excluded from this dataset were patients with any previous cancer, except for basal-cell carcinoma of the skin, as well as those whose first primary cancer diagnosis and death were simultaneously recorded.

*Second primary cancer data*. Subsequent SPCs among PC patients were retrieved. The FCR does not record cases of SPCs occurring at the same site as the index tumour, such as oropharynx-oropharynx, unless the cancer is of a different histological type. Still, it is essential to acknowledge the potential for a minimal degree of misclassification, wherein certain regional or distant recurrences might be erroneously categorized as SPCs, and vice versa. To mitigate this potential misclassification, only metachronous SPCs – malignant tumours diagnosed six months or more after the initial diagnosis of primary PC – were included. Synchronous SPCs – diagnosed within six months of the primary PC diagnosis – were thus excluded (*n* = 72). Follow-up started upon diagnosis of primary PC and concluded at the time of death, diagnosis of a second primary cancer (SPC), or December 31st 2021; whichever event occurred first. None of the patients emigrated during the follow-up period.

*Statistical analyses*. To estimate the risk of SPC, standardized incidence ratios (SIRs) and their associated 95% confidence intervals (CIs) were calculated for each pharyngeal subsite through a comparison between the observed number of SPC cases among PC patients and the expected numbers. The expected values were derived from age, sex, and calendar-specific cancer rates in the Finnish general population, employing PYs of observations (up to first primary cancer) and assuming a Poisson distribution for the observed cases. SIRs were stratified by age at diagnosis, sex, extent of the primary disease (localized or non-localized), time elapsed since primary cancer diagnosis, and calendar period of PC diagnosis (1953–1989, 1990–2005, 2005–2021) to investigate whether the risk of SPCs varied over the follow-up period. The excess absolute risk (EAR) was calculated to assess the absolute measure that quantifies the difference in absolute risk between the PC patient population and the general population, expressed as the number of excess cancers per 1000 person-years (PYs) at risk. In simpler terms, EAR provides insight into the absolute number of additional cancers attributable to primary PC, thereby evaluating the additional burden of SPCs. For data privacy reasons, observed numbers and risk estimates are not shown, whenever less than five cancer cases were reported. All the statistical analyses were conducted using R software (The R Project for Statistical Computing), version 4.2.2, and involved the utilization of the *popEpi* and *forestplot* packages.

## Results

A total of 4701 PC patients – 3320 men (71%) and 1381 women (29%) – were identified from 1953 to 2021 adding to 25 215 PYs of follow-up. The average and median follow-up times were 5.7 and 2.8 years, respectively (first quartile 0.82 years, third quartile 8.07 years). The oropharynx, nasopharynx, and hypopharynx contributed to 61.2% (*n* = 2 879), 14.3% (*n* = 672), and 21.3% (*n* = 1 003) of all cases, respectively. A total of 147 cases (3.1%) were recorded in a non-specified pharyngeal site. Of all cases, 88.8% were SCCs.

**Fig. 1:**
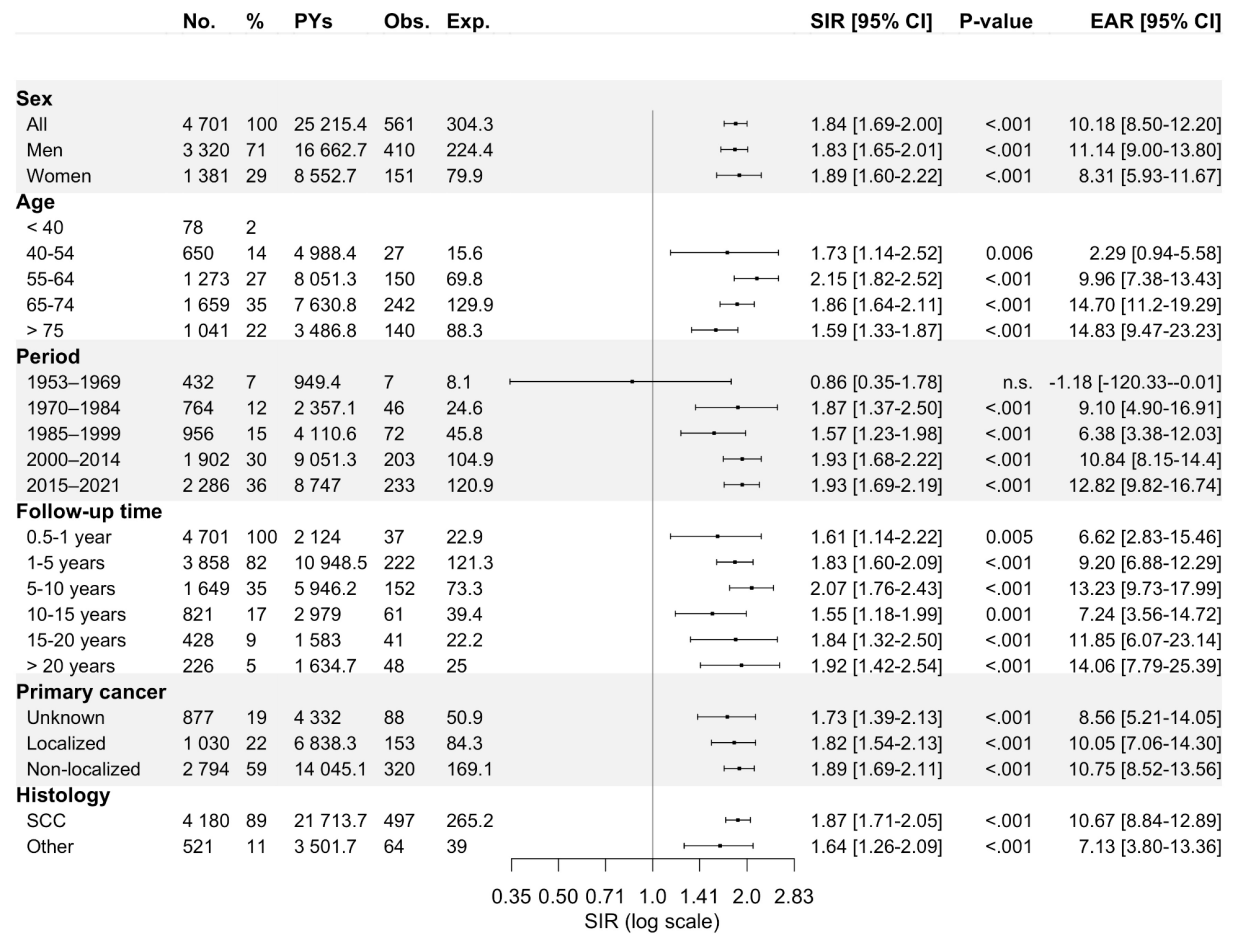
Standardized incidence ratios and excess absolute risk per 1 000 person-years for any metachronous second primary cancer among 4 701 **pharyngeal carcinoma** patients diagnosed in Finland during 1953–2021 stratified by sex, age at diagnosis of primary tumour, follow-up period, follow-up time, primary disease stage, and histology. Abbreviations: CI, confidence interval; Exp., expected; EAR, excess absolute risk; n.s., non-significant; No., number; Obs., observed; PYs, person-years; SCC, squamous cell carcinoma; SIR, standardized incidence ratio

A metachronous SPC was diagnosed in 561 patients (11.9% of all PC patients) over the entire follow-up: in 12.3% of men (*n* = 410) and 10.9% of women (*n* = 151). The respiratory and digestive organs harboured most SPCs, with 25.5% (*n* = 143) and 21.4% (*n* = 120) of all diagnosed SPCs, respectively Out of all SPCs, 39.6% were diagnosed within 0.5 to 5 years and 26.7% after 10 years from the initial PC diagnosis (Fig. 1). For male PC patients, the overall SIR for an SPC at any primary site was 1.83 (95% CI: 1.65–2.01). Similarly, for female patients, the corresponding SIR was 1.89 (95% CI: 1.60–2.22).

**Fig. 2:**
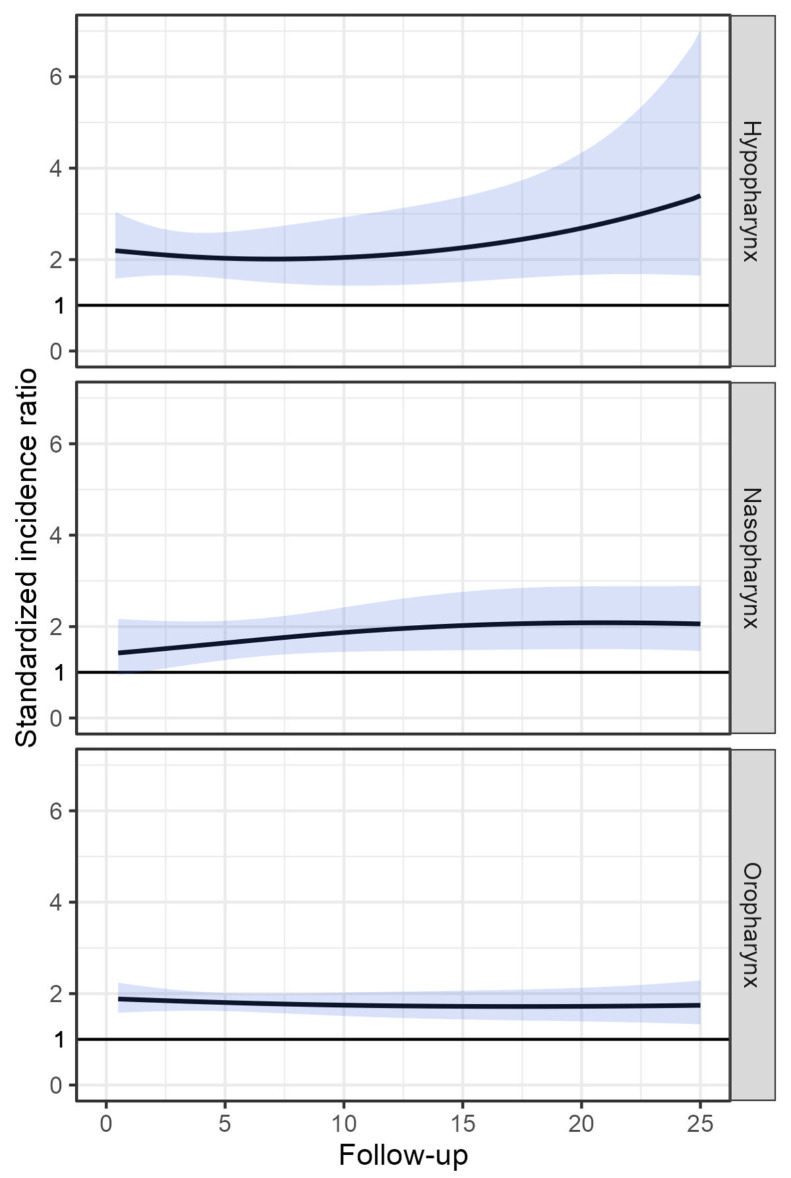
Risk of second primary cancer among **pharyngeal carcinoma** patients as a function of follow-up time and by primary cancer site

The risk of SPC remained elevated in all age categories over 40, in each calendar period after 1970, for both localized and non-localized primary disease, and for primary pharyngeal SCC and other primary carcinomas, when compared to the cancer risk in the general population. Additionally, following diagnosis of PC, the risk of SPC persisted throughout the follow-up, even after 20 years. Figure 2 illustrates the risk of SPC as a function of follow-up time stratified by subsite of the first primary PC.

**Fig. 3:**
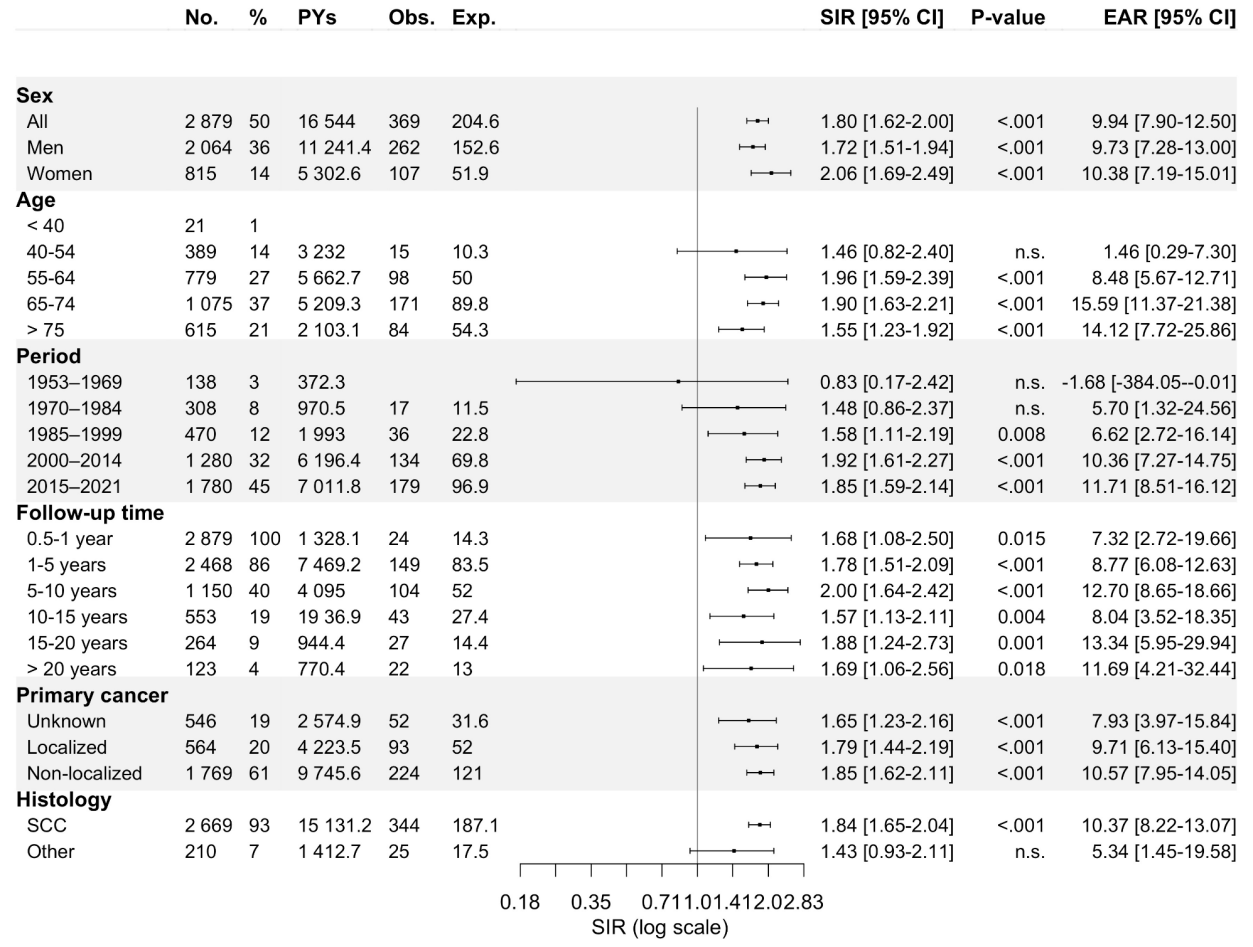
Standardized incidence ratios and excess absolute risk per 1 000 person-years for any metachronous second primary cancer among 2 879 oropharyngeal carcinoma patients diagnosed in Finland during 1953–2021 stratified by sex, age at diagnosis of primary tumor, follow-up period, follow-up time, primary disease stage, and histology. Abbreviations: CI, confidence interval; Exp., expected; EAR, excess absolute risk; n.s., non-significant; No., number; Obs., observed; PYs, person-years; SCC, squamous cell carcinoma; SIR, standardized incidence ratio

**Fig. 4:**
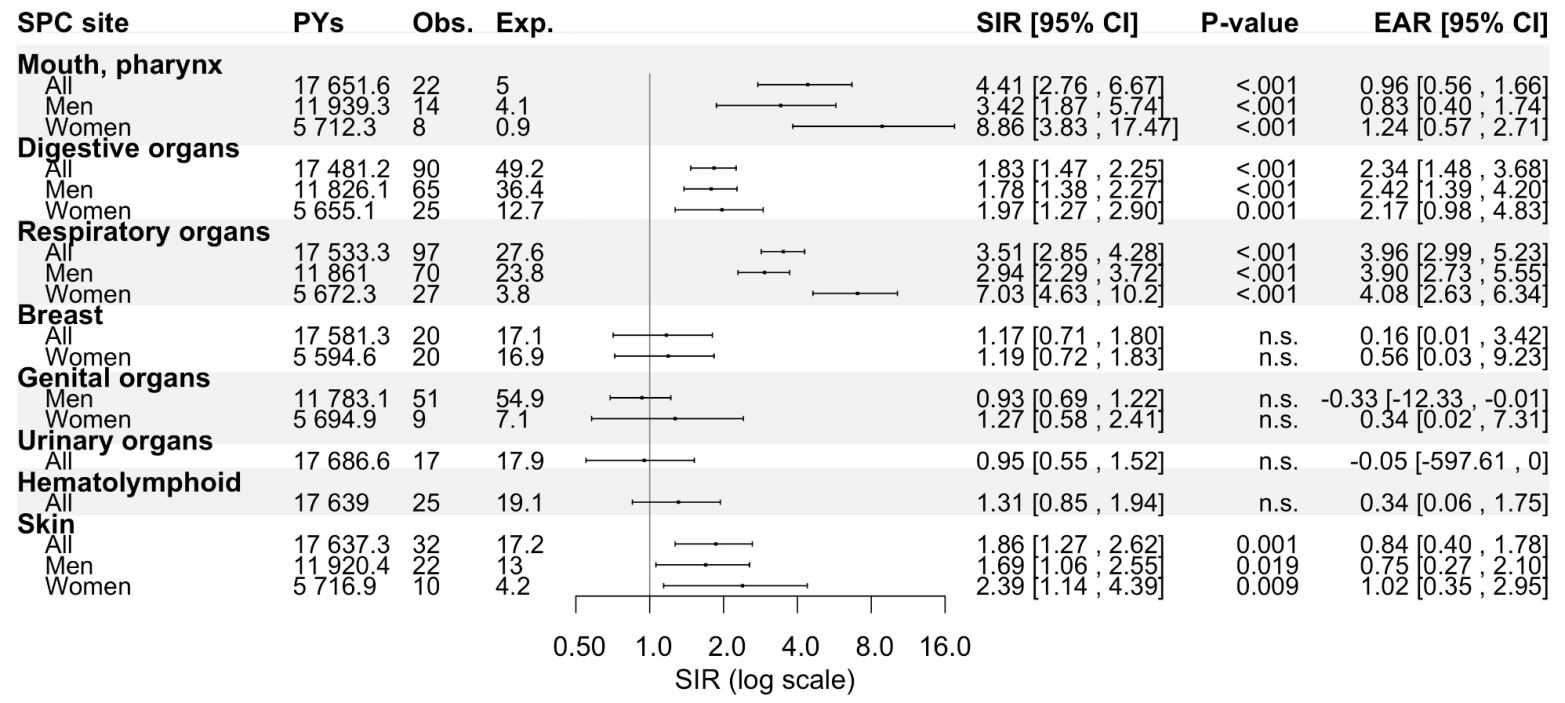
Standardized incidence ratios and excess absolute risk per 1 000 person-years for second primary cancer by site (if more than four cases recorded) among patients with oropharyngeal carcinoma in Finland during 1953–2021. Abbreviations: CI, confidence interval; Exp., expected; EAR, excess absolute risk; n.s., non-significant; No., number; Obs., observed; PYs, person-years; SIR, standardized incidence ratio

*Oropharynx.* Of all OPC patients (92.3% SCCs), a metachronous SPC was diagnosed in 12.7% (*n* = 262) and 13.1% (*n* = 107) of men and women, respectively, which translated to SIRs of 1.72 (95% CI: 1.51–1.94) and 2.06 (95% CI: 1.69–2.49) (Fig. 3). The overall risk of SPC remained elevated even after 20 years of follow-up. The risk of SPC was specifically elevated for oropharyngeal SCC (OPSCC) but not for other types of carcinomas. However, when stratified by SPC site, the risk of SPC in digestive organs was notable only during the initial 10 years of follow-up. OPC patients displayed increased SIRs for cancer of the mouth/pharynx (SIR 4.41, 95% CI: 2.76–6.67), digestive organs (1.83, 1.47–2.25), respiratory organs (3.51, 2.85–4.28), and skin (1.86, 1.27–2.62), as shown in Fig. 4. Regarding the respiratory organs, women exhibited a higher SIR for SPCs than men (7.03, 95% CI: 4.63–10.2 versus 2.94, 2.29–3.72).

**Fig. 5:**
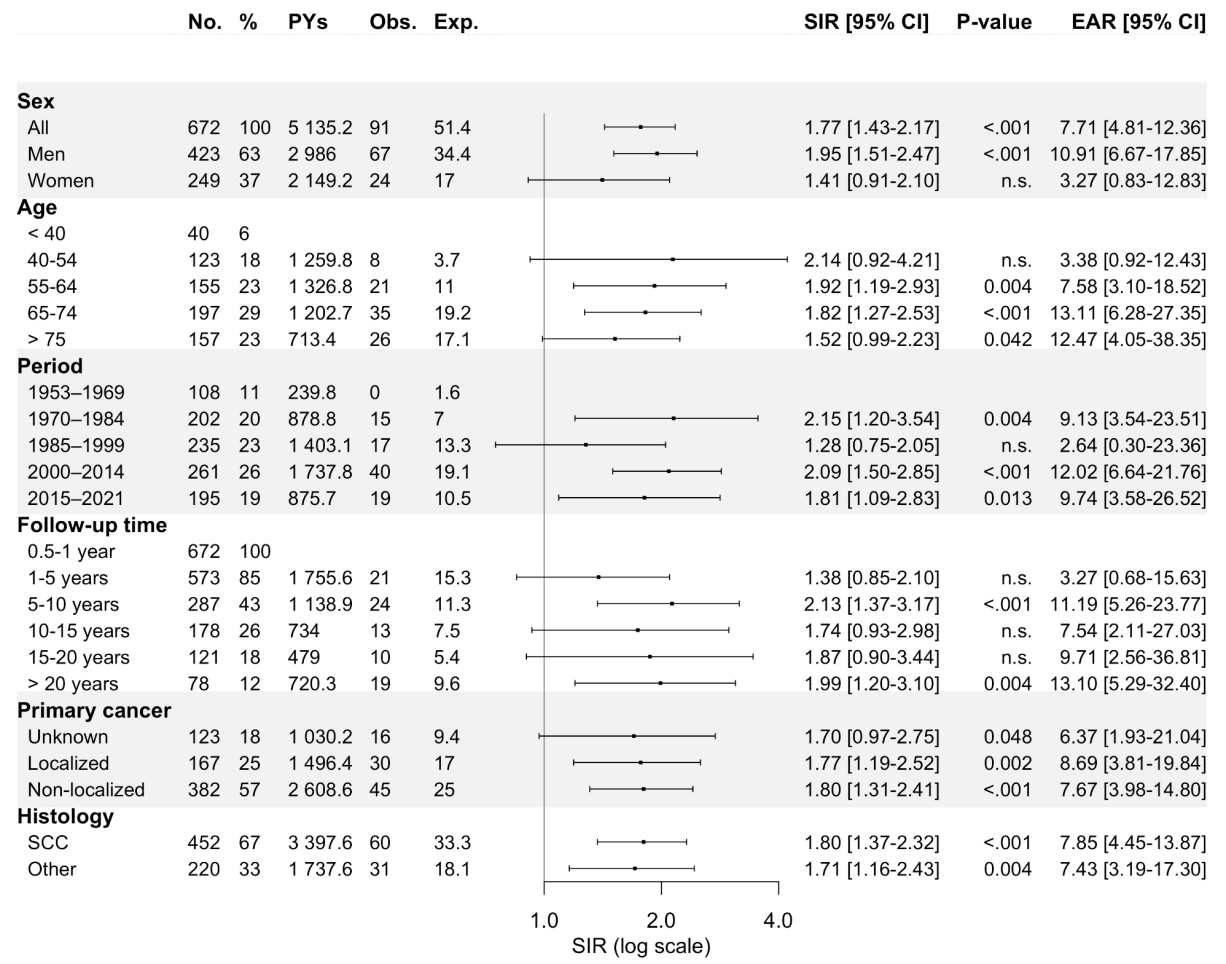
Standardized incidence ratios and excess absolute risk per 1 000 person-years for any metachronous second primary cancer among 672 nasopharyngeal carcinoma patients diagnosed in Finland during 1953–2021 stratified by sex, age at diagnosis of primary tumor, follow-up period, follow-up time, primary disease stage, and histology. Abbreviations: CI, confidence interval; Exp., expected; EAR, excess absolute risk; n.s., non-significant; No., number; Obs., observed; PYs, person-years; SCC, squamous cell carcinoma; SIR, standardized incidence ratio

**Fig. 6:**
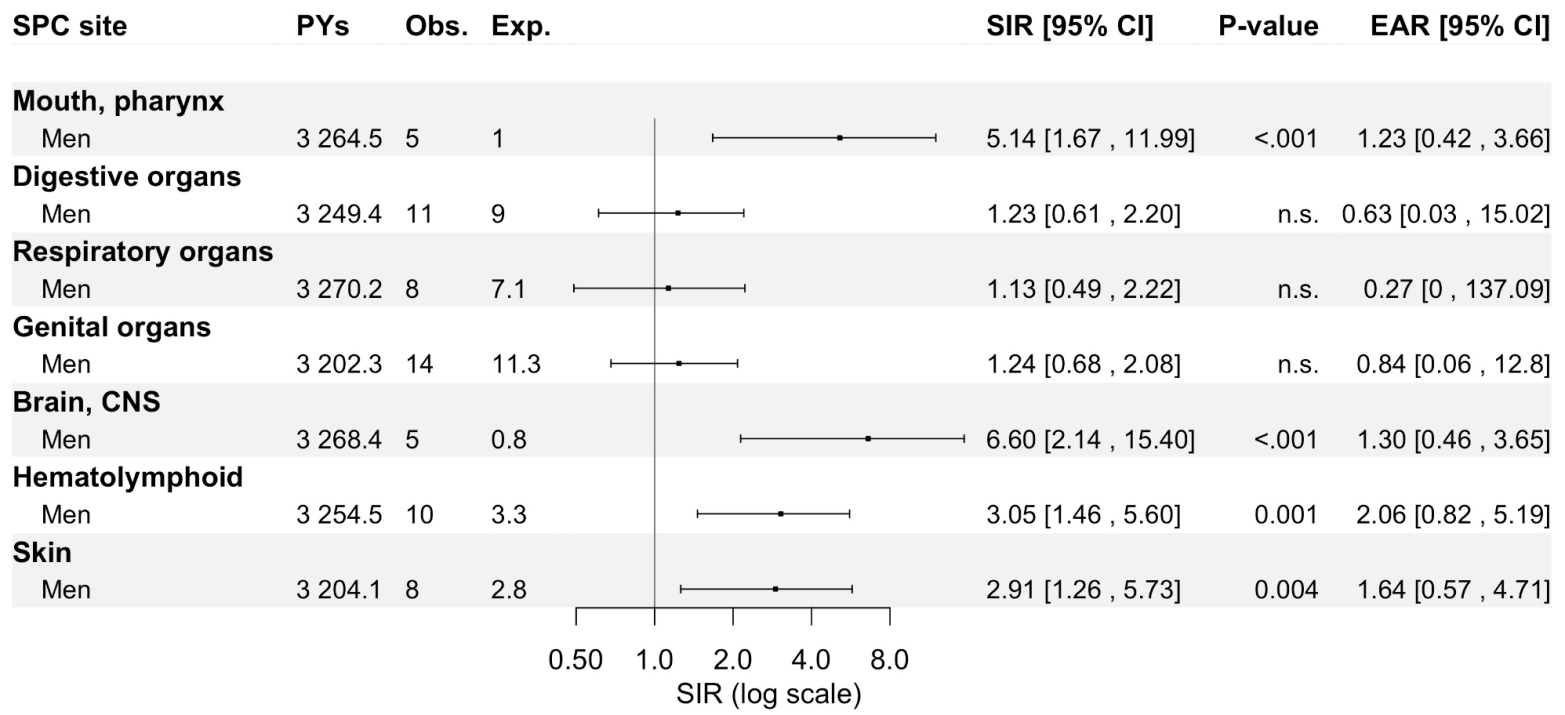
Standardized incidence ratios and excess absolute risk per 1 000 person-years for second primary cancer by site (if more than four cases recorded) among men with nasopharyngeal carcinoma in Finland during 1953–2021. Abbreviations: CI, confidence interval; Exp., expected; EAR, excess absolute risk; n.s., non-significant; No., number; Obs., observed; PYs, person-years; SIR, standardized incidence ratio

*Nasopharynx.* Of all NPC patients (68.0% SCCs), a metachronous SPC was diagnosed in 14.8% (*n* = 63) and 8.6% (*n* = 37) of men and women, respectively (Fig. 5). An excess risk of SPC was seen among men (SIR 1.95, 95% CI: 1.51–2.47), observable even after 20 years of follow-up. Men displayed increased SIRs for cancers of the mouth/pharynx (SIR 5.14, 95% CI: 1.67–11.99), brain (6.60, 2.14–15.40), haematolymphoid tissues (3.05, 1.46–5.60), and skin (2.91, 1.26–5.73), as shown in Fig. 6. Conversely, women did not show an increased risk of SPC for any site. The risk for haematolymphoid cancers among men was solely increased during the initial five years of follow-up (SIR 3.83, 95% CI: 1.24–8.93) and specifically among patients aged over 75-years (SIR 3.33, 95% CI: 1.22–7.24).

**Fig. 7:**
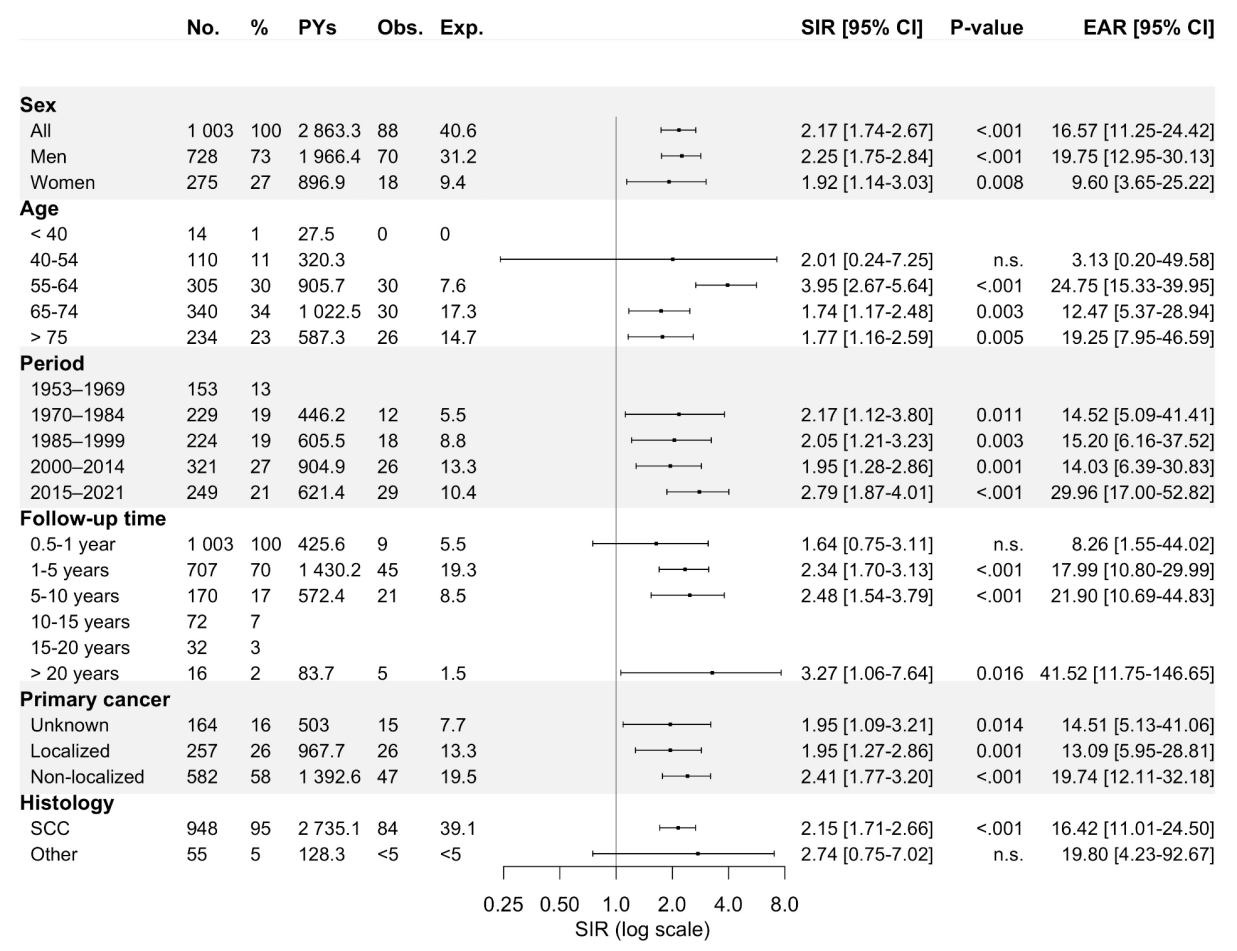
Standardized incidence ratios and excess absolute risk per 1 000 person-years for any metachronous second primary cancer among 1 003 hypopharyngeal carcinoma patients diagnosed in Finland during 1953–2021 stratified by sex, age at diagnosis of primary tumor, follow-up period, follow-up time, primary disease stage, and histology. Abbreviations: CI, confidence interval; Exp., expected; EAR, excess absolute risk; n.s., non-significant; No., number; Obs., observed; PYs, person-years; SCC, squamous cell carcinoma; SIR, standardized incidence ratio

**Fig. 8:**
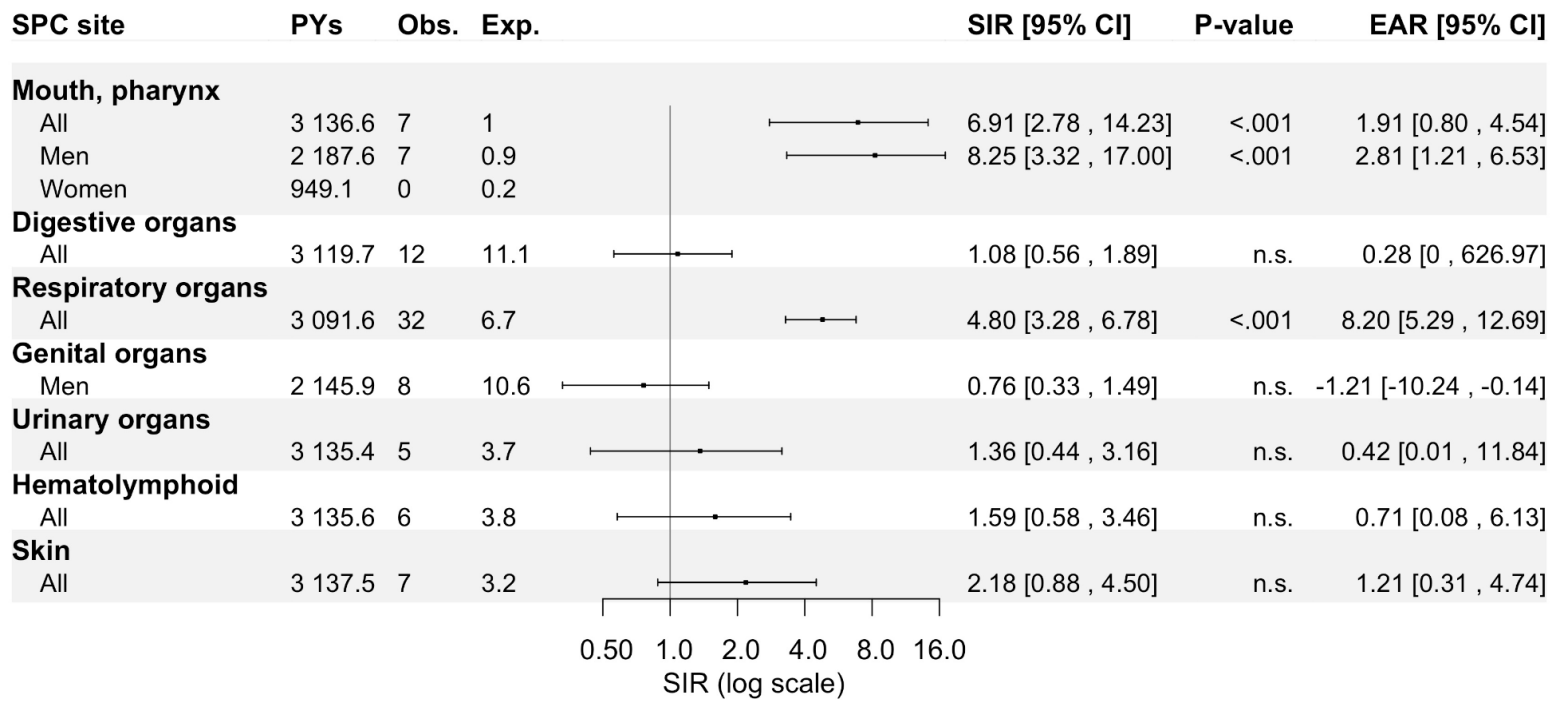
Standardized incidence ratios and excess absolute risk per 1 000 person-years for second primary cancer by site (if more than four cases recorded) among patients with hypopharyngeal carcinoma in Finland during 1953–2021. Abbreviations: CI, confidence interval; Exp., expected; EAR, excess absolute risk; n.s., non-significant; No., number; Obs., observed; Pys, person-years; SIR, standardized incidence ratio

*Hypopharynx.* Of all hypopharyngeal carcinoma (HPC) patients (94.5% SCCs), a metachronous SPC was diagnosed in 10% of men (*n* = 73) and 9.8% of women (*n* = 27), which corresponded to SIRs of 2.25 (95% CI: 1.75–2.84) and 1.92 (95% CI: 1.14–3.03), respectively (Fig. 7). As for other pharyngeal sites, the overall risk of SPC remained elevated over the entire follow-up period. The increased risk of SPC was observed only for hypopharyngeal SCC (HPSCC) and not for other carcinomas. Men displayed increased SIRs for cancers of the mouth/pharynx (SIR 8.25, 95% CI: 3.32-17.00) and respiratory organs (4.61, 3.06–6.66), as shown in Fig. 8. Women displayed an elevated risk only for cancers of the respiratory organs, however, fewer than five cases were recorded. Of note, although no increased risk of SPC was observed for the digestive organs overall, an in increased risk of oesophageal cancer (not shown) was identified among both men and women (SIR 5.87, 95% CI: 1.60-15.04). However, fewer than five cases were diagnosed.

## Discussion

The current study confirms that patients with a history of PC carry an overall excess risk of over 80% for developing an SPC compared to the general Finnish population. Our findings further indicate that this elevated SPC risk persists even beyond 20 years following the diagnosis of primary PC (SIR 1.92, 95% CI: 1.42–2.54 after 20 years). In addition to an increased cancer risk in the mouth/pharynx and respiratory organs observed among OPC (SIR 4.41 and 3.51, respectively) and HPC patients (SIR 6.91 and 4.80), an elevated cancer risk in digestive organs was noted among OPC patients (SIR 1.83). Apart from the aerodigestive organs and skin, the risk of SPC was not elevated for other anatomical sites among these patients. Male NPC patients exhibited an increased risk for oral/pharyngeal, brain, haematolymphoid, and skin cancers (SIRs 5.14, 6.60, 3.05, and 2.91, respectively).

The risk of SPC in the mouth/pharynx, respiratory, and digestive organs among OPC and HPC patients aligns with results from previous studies and may be explained by the field cancerization effect induced by the chronic use of tobacco and alcohol drinking [11, 12]. Supporting our findings, Morris et al. [13] conducted a study in the US utilizing data from the Surveillance, Epidemiology, and End Results (SEER) database. They analysed 75 087 cases of HNC and reported SIRs of 2.99 (95% CI: 2.88–31.10) and 3.47 (3.27–3.68) for OPSCC and HPSCC, respectively, for any SPC. The study, which had a median follow-up time of 5.8 years, identified elevated risks not only in the mouth/pharynx, respiratory, and digestive organs, but also observed an excess of lymphoma and thyroid cancer among OPSCC and HPSCC patients, respectively. In South Korea, Jung et al. [14]. reviewed data of 5587 OPSCC and 3685 HPSCC patients with mean follow-up periods of 4.2 and 2.6 years, respectively. The authors conveyed SIRs of 1.46 (95% CI: 1.30–1.63) and 1.93 (1.71–2.17) for any SPC among OPSCC and HPSCC patients, respectively, with elevated SPC risks in the mouth/pharynx, respiratory organs, and oesophagus.

In our study the risk of SPC among OPC patients remained elevated in each time period after 1985 with no major differences observed. The prevalence of HPV in OPC has significantly increased over time worldwide. For instance, in Europe, HPV-positive OPC rose from 39.7% (95% CI: 32.8-47.0) before 2000 to 59.0% (95% CI: 30.2-82.7) between 2000 and 2004 [15]. The average latency period from infection to HPV-positive OPC is estimated at around 10 to 30 years. Thus, the SIRs could change with longer follow-up [16].

Women exhibited higher SIRs for most tobacco and alcohol associated SPC sites, especially among OPC patients for SPCs occurring in the respiratory organs aligning with previous studies [17–20]. Smoking and alcohol use influence the development of SPCs in the upper aerodigestive tract [21, 22], and the observed higher risk for a SPC in women, as described also in previous studies, may be attributable to the intrinsic nature of the SIR analysis, where the observed incidence is compared with the expected number derived from age, sex, and calendar-specific rates in the general population. Given the higher prevalence of smoking and alcohol and the incidence of related diseases in men compared to women [23, 24], the SIRs are consequently higher in women due to the lower rates in the reference population.

In our study 9.7% of NPC patients were diagnosed with an SPC within the first 5 years of follow-up and 12% during the first 10 years. A recent systematic review [10], encompassing 21 articles – among others two from Europe, three from the USA, and 11 from China, Hong Kong, Singapore, and Taiwan – reported a 6.6% occurrence rate (range 1.5–20.2) of SPC with an average follow-up time of 7.7 years. The overall SIR of SPC for all countries and cancer sites combined was 2.0, consistent with our risk estimate. NPC is typically treated with high-dose radiotherapy combined with chemotherapy, commonly cisplatin [25]. The elevated risk of brain cancer observed among NPC patients could be subsequent sequelae from radiotherapy, as the brain would be exposed to the radiation [26]. However, the advent of intensity-modulated radiotherapy (IMRT) as the standard technique for NPC treatment has not only enhanced disease control but also minimized unintended radiation to the brain [27]. IMRT was introduced in the late 1990s and became widely adopted in Finland in the early 2000s. Within our patient cohort, we observed five cases of brain SPCs, with four diagnosed before the widespread use of IMRT, lending support to the hypothesis that radiation played a role in their development. Still, there is also a chance of mistakenly classifying an extensive nasopharyngeal NPC as brain cancer.

Our study reveals an increased incidence of skin cancer after five years among patients with OPC and NPC, mirroring findings in Taiwan where a heightened risk of skin cancer was noted among NPC patients [19]. It could be argued that PCs are not inherently linked to UV radiation exposure, and plausible that shared lifestyle factors, such as outdoor activities, among these patients may contribute to the elevated risk of skin cancer. However, in Nordic countries, where outdoor occupations have not been associated with an increased risk of OPC or NPC cancers, this explanation may not entirely apply [28, 29]. It would make more sense that the elevated skin cancer risk would also be attributable to radiation exposure, which can increase the risk of skin cancer in the irradiated area [30].

Concerning the risk of haematolymphoid cancers among NPC patients, case reports delineate instances of these cancers diagnosed after chemoradiotherapy treatment [31, 32]. Cisplatin has been associated with an elevated risk of secondary leukaemia following treatment of ovarian and testicular cancers [33, 34]. Occupational exposure to specific agents may also contribute to this risk. Indeed, evidence, albeit weak, suggest a link between formaldehyde exposure and NPC, leukaemia, and non-Hodgkin’s lymphoma [35]. Additionally, common genetic susceptibility may play a role in these associations. For instance, polymorphisms in the DNA repair gene XRCC1 have been associated with an elevated risk of both NPC and leukaemia in specific populations [36, 37]. Lastly, the heightened risk of haematolymphoid cancers described may simply reflect enhanced surveillance and detection of these malignancies, exemplified in our study by the elevated risk of haematolymphoid cancers exclusively observed among male patients aged 75 years or older during the initial five years of follow-up.

Studies investigating the risk of SPC following primary PC in Europe have typically relied on small case series, with very few exceptions [2, 3, 10]. To the best of our knowledge, the present cohort represents one of the largest published in Europe thus far with the most extensive follow-up. The underreporting of cancers in the FCR is negligible, as quality assessment studies have demonstrated high coverage (95% for head and neck tumours in 2009–2013) and diagnostic accuracy [38]. One of the major strengths of this study lies in its low risk of misclassifying a recurrence as an SPC, which is attributable to the FCR’s practice of not documenting subsequent cancers that occur at the same site as the index site. Thought, this could also lower the overall SPC risk. Moreover, population-based studies help mitigate selection bias inherent in hospital or clinical series [39].

Within the context of cancer register-based studies, it is crucial to consider the inherent limitations of our results. The absence of data on aetiological factors like tobacco, alcohol consumption, and HPV status, as well as details about primary treatment, especially radio- and chemotherapy regimens, hinders our ability to draw conclusions about their relationship to SPCs. Moreover, assessing the specific impact of HPV vaccination on SPC risk – particularly in patients with OPSCC – will require dedicated evaluation in the future [40]. Additionally, our study is susceptible to misclassification of the exact site, an unfortunate reality in clinical practice. The proximity of some SPC sites to the primary tumour could have led to misdiagnoses of local spread or disease recurrence as SPCs and primary tumour metastases might have been inaccurately classified as SPCs and vice versa. However, this potential diagnostic bias cannot solely explain the association between PC and increased SPC risk, as the risk remained elevated even beyond 20 years post-diagnosis of the primary tumour.

## Conclusions

In summary, in Finland, mirroring previous findings from other countries, individuals diagnosed with PC face an 80% higher risk of developing an SPC compared to the cancer risk of the general population of same age and sex, and this elevated risk persists beyond 20 years following the diagnosis of the initial cancer. Notably, strong associations were observed between OPC/HPC and SPCs related to tobacco/alcohol, such as cancers of the mouth/pharynx and lungs, suggestive of shared risk factors for the development of multiple cancers. Male patients with NPC not only experience an elevated risk of cancer in the mouth/pharynx but also face an increased risk for haematolymphoid cancers, possibly stemming from treatment-related side effects. Moreover, OPC and NPC patients carry an increased risk of skin cancer, likely attributed to the delayed effects of radiotherapy. Healthcare professionals must be mindful of the SPC risk in survivors of PC who should receive counselling about this phenomenon and be encouraged to adopt a healthy lifestyle, including smoking cessation while remaining vigilant about symptoms, also beyond the usual 5-year follow-up period.

## Data Availability

Raw data sharing not available due to privacy restrictions.

## Abbreviations

CI: Confidence Interval
EAR: Excess Absolute Risk
FCR: Finnish Cancer Registry
HNC: Head and Neck Cancer
HPC: Hypopharyngeal Carcinoma
HPSCC: Hypopharyngeal Squamous Cell Carcinoma
HPV: Human Papillomavirus
IMRT: Intensity-Modulated Radiotherapy
NPC: Nasopharyngeal Carcinoma
OPC: Oropharyngeal Carcinoma
OPSCC: Oropharyngeal Squamous Cell Carcinoma
PC: Pharyngeal Carcinoma
PY: Person-Year
SCC: Squamous Cell Carcinoma
SIR: Standardized Incidence Ratio
SPC: Second Primary Carcinoma
